# Associations of cigarette smoking and burden of thoracic aortic calcification in asymptomatic individuals: A dose-response relationship

**DOI:** 10.1371/journal.pone.0227680

**Published:** 2020-01-09

**Authors:** Jui-Peng Tsai, Ya-Ting Jan, Chun-Ho Yun, Kuo-Tzu Sung, Chuan-Chuan Liu, Jen-Yuan Kuo, Chung-Lieh Hung, Tung-Hsin Wu, Jiun-Lu Lin, Charles Jia-Yin Hou, Hung-I Yeh, Hiram G. Bezerra, Aaron So

**Affiliations:** 1 Department of Biomedical Imaging and Radiological Sciences, National Yang Ming University, Taipei, Taiwan; 2 Division of Cardiology, Department of Internal Medicine, MacKay Memorial Hospital, Taipei, Taiwan; 3 Department of Medicine, Mackay Medical College, Taipei, Taiwan; 4 Mackay Junior College of Medicine, Nursing and Management, Taipei, Taiwan; 5 Department of Radiology, MacKay Memorial Hospital, Taipei, Taiwan; 6 Graduate Institute of Health Care Organization Administration, College of Public Health National Taiwan University, Taipei, Taiwan; 7 Health Evaluation Center, MacKay Memorial Hospital, Taipei, Taiwan; 8 Department of Medical Technology, Yuanpei University of Science and Technology, Hsin-Chu, Taiwan; 9 Division of Endocrinology and Metabolism, Department of Internal Medicine, MacKay Memorial Hospital, Taipei, Taiwan; 10 Cardiovascular Department, University Hospitals Case Medical Center, Cleveland, OH, United States of America; 11 Imaging Program, Lawson Health Research Institute, London, Ontario, Canada; 12 Department of Medical Biophysics, University of Western Ontario, London, Ontario, Canada; Beijing Key Laboratory of Diabetes Prevention and Research, CHINA

## Abstract

Smoking is known as a powerful predictor of pathological coronary atherosclerosis. Thoracic aortic calcification (TAC), an alternative marker for pathological atherosclerosis, has also been shown to be associated unfavorable cardiovascular outcomes. We aimed to investigate the dose-response relationship between cigarette use and calcification burden in subjects free from clinical symptoms. Among 3109 patients enrolled in this analysis, we categorized study participants according to smoking exposure pattern as: non-smokers, ex-smokers and current smokers. Smoking dose (cigarette/day), duration (years) and pack-years were semi-quantified as smoking dose exposure variables. Thoracic aortic calcification burden (including TAC score, plaque volume and plaque density) were determined and related to smoking dose and pattern information. TAC burdens (including TAC score, plaque volume and density) were highest in current smoker compared to non-smoker group, with ex-smoker showing TAC burdens in-between (all ANOVA p<0.05). Linear regression models consistently demonstrated that TAC burdens as continuous variables were independently higher in a dose-dependent manner with smoking exposure, particularly in high-dose (> 10 cigarettes/day) and the long-duration (> 3 years) smokers, even after adjusting for baseline demographic differences (all p<0.05). By logistic regression, subjects who never smoke consistently demonstrated reduced risk of TAC existence (adjusted OR: 0.65 [95% CI: 0.48–0.86], P = 0.003) in contrary to those current smokers (adjusted OR: 1.47 [95% CI: 1.10–1.89], P = 0.009). A dose-response relationship between active cigarette use and TAC burden was observed, with those who never exposed to smoking or quitted demonstrating partial protective effects. Our data provided imaging-based evidence about the potential deleterious biological hazards of long-term and high-dose cigarette consumption.

## Introduction

Atherosclerosis is the primary cause of coronary artery disease (CAD) and the plaque formation in the coronary arteries is mediated by the oxidative low-density lipoprotein (LDL) particles and inflammatory cytokines resided in the coronary endothelium.[1] Coronary artery calcification (CAC) is a surrogate marker of atherosclerosis and strong predictor of CAD.[2] There exists a positive and independent correlation between the CAC volume measured by computed tomography (CT) and the risk of CAD.[3] Accumulating data has shown that thoracic aortic calcification (TAC) and CAC are both tightly associated with incident cardiovascular events.[4] In addition, calcified depots in thoracic aorta and anatomic heart valve regions have been shown to be strong indicator of systemic vascular disease.[5]

Smoking is the one of leading preventable causes of cardiovascular disease (CVD) and contributes to approximately 30% of the CVD related death worldwide.[6] Smoking-related cardiovascular toxicity is associated with inflammation, endothelial dysfunction, reduced nitric oxide (NO) bioavailability, and platelet aggregability.[7,8] A significant dose-response relationship between cigarette smoking and atherosclerosis[9,10] and subclinical coronary atherosclerosis[11] has been reported in previous studies. Additionally, smoking may further increase the risk of aortic arch calcification in patients with a Chinese ethic background.[7] Although the risk of coronary heart disease (CHD) significantly decreases after cessation of smoking, it remains significantly higher than that of non-smokers for many years, suggesting the possibility of an irreversible damage mediated by coronary and aortic calcifications.[12]

The objective of this study was to examine the correlation between cigarette consumption and severity of plaque calcification in the thoracic aorta in Chinese smokers, with the plaque burden evaluated based on the TAC score, plaque volume and plaque density measured by multi-detector computed tomography (MDCT).

## Materials and methods

### Study population

From Jan 2005 to Dec 2012, a total of 3109 consecutive subjects were enrolled from a single-center (MacKay Memorial Hospital) for this retrospective study. The study complies with the Declaration of Helsinki, and was approved by the Institutional Review Board of Mackay Memorial Hospital, Taipei, Taiwan (17MMHIS082e, Form of written consent was waived by IRB). All data were analyzed retrospectively and de-identified prior to author access and analysis. The study participants underwent annual cardiovascular health survey received non-contrast MDCT for the assessment of cardiovascular risk by assessing coronary artery calcium scores. All subjects were clinically symptom free from chest pain, angina or dyspnea and first divided into three study groups according to their smoking history: non-smokers (n = 2234), ex-smokers (n = 86), and current smokers (n = 782). The current smokers were further divided into three categories according to their smoking habits: smoking dose, smoking duration and total pack-years.[9] Smoking dose was further stratified into three levels: 1–10 cigarettes/day (light smoker), 11–20 cigarettes/day (moderate smoker), > 1 pack/day (heavy smoker); Similarly, smoking duration were further stratified into five levels: < 1 year; 1–3 years, 3–5 years, 5–10 years, > 10 years.

### Thoracic aortic and coronary calcium measurement

MDCT of the heart was performed using a 16-slice scanner (Sensation 16, Siemens Medical Solutions, Forchheim, Germany) with 16 x 0.75 mm collimation, 420ms rotation time, tube voltage of 120 kV. Patients were instructed to hold their breath during the CT acquisition. All images were acquired from above the level of tracheal bifurcation to below the base of the heart using prospective ECG triggering with the center of acquisition set at 70% of the R-R interval as previously described.[11] The TAC scores and coronary calcium burden as coronary calcium score (CCS) were derived from the cardiac CT images based on the method previously described.[13] Calcification in the thoracic aorta and coronary vasculature were defined as the plaque region with a density > 130 HU. The total coronary calcium burden expressed as CCS (n = 3084) was quantified utilizing same workstation and expressed as Agatston scores. To determine the TAC score, plaque area (mm^2^) lesion areas with a density of 130~199 HU were multiplied by 1, those with 200~299 HU were multiplied by 2, those with 300~399 HU were multiplied by 3, and those with 400 HU or greater were multiplied by 4. These scores are therefore upweighted for increased plaque density. The total plaque-specific score was calculated by adding the score of each slice.

**Fig 1:**
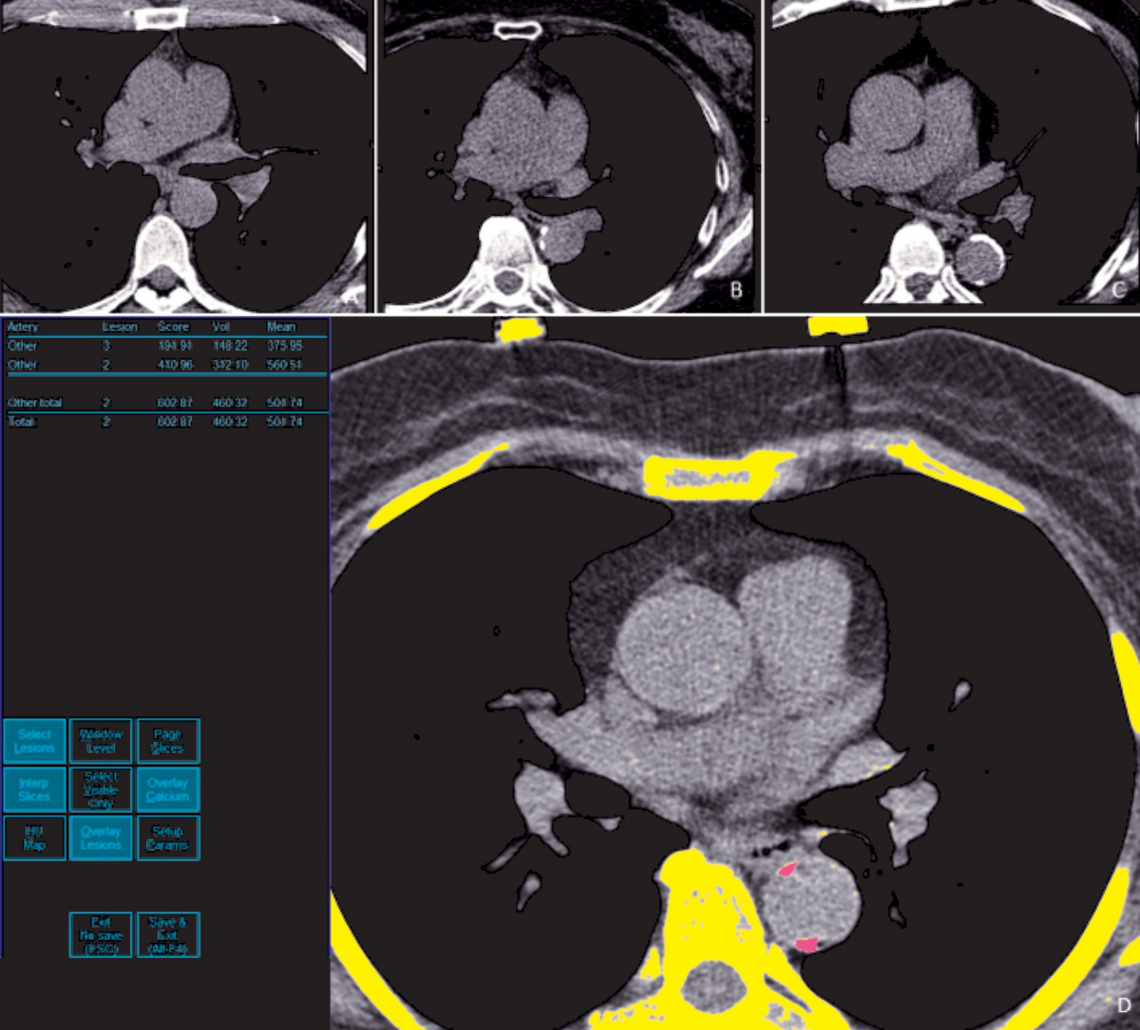
Illustration of thoracic aortic calcification analysis in current study. Axial images of ECG-gated, non-enhanced cardiac CT studies with no calcified plaque in a nonsmoker (a), mild calcified plaques in a light smoker and moderate calcified plaques in a heavy smoker (c). Thoracic aortic calcification analysis and software analysis illustrations (d) of aorta and sites of calcification chosen by the software demonstrated as “pink” color as labelled aortic calcified plaques (right-sided CT axial image). Results of Agaston score (TAC) (602.9), total plaque volume (460.3mm^3^) and mean density (501.7 HU) of total plaques (left-sided column) were displayed automatically by the software.

To calculate volume scores, plaque volume (mm^3^) was determined by multiplying the sum of all plaque areas by the CT slice thickness. All image analyses were performed using a dedicated workstation (Aquarius 3D Workstation, TeraRecon, San Mateo, CA, USA), which also provided the parameter of “TAC density” as the mean density in Hounsfield unit of all selected calcified plaques in the process of TAC score analysis. (**Fig 1**)

### Statistical analysis

Continuous variables were presented as mean ± standard deviations (SDs); categorical variables were presented as numbers or percentages. One-way analysis of variance (ANOVA) was performed to determine if there existed statistically significant difference in the mean values of aortic calcification data among groups (smoking dose and years smoking), with post-hoc tests used for pair-wise comparisons, with chi squared test used to compare differences of categorical variables among groups.

Uni-variate and multi-variate linear regression models were constructed to evaluate the associations between smoking doses and 3 aortic calcification measures as continuous variables, including volume scores, plaque volume and TAC density, separately. Multi-variate linear regression analyses were adjusted for baseline conventional atherosclerotic risk profiles including age, sex, body mass, blood pressure, heart rate, lipid profiles and medical histories of hypertension, diabetes and known cardiovascular diseases. For smoking does, the lowest smoking dosage (in years, cigarettes/d or pack-years in different models) served as reference group for regression models. As aging, obesity, higher blood pressure, and worsened renal function have been proposed as key factors driving aortic calcification, we further tested their modifying effects with smoking doses in the presence of aortic calcification (as binary variable).[14–16]

To further explore the associations of different smoking exposure patterns (including current, ex-, and non-smokers) and presence of aortic calcification as outcome measure (as binary variable), we further conducted models by using logistic regression analyses (data presented with odds ratios [ORs] and 95% confidence interval) to establish the link of distinct smoking history and development of aortic calcification.

All statistical analyses were performed using SPSS software (IBM Corp, Armonk, NY) and Stata version 14 (Stata Corp., College Station, TX) and SAS 9.2 version (SAS Institute, Cary, North Carolina), and a two-tailed p value < 0.05 indicated statistical significance.

## Results

### Participant demographics and clinical characteristics

Participant demographics and clinical characteristics categorized by smoking habits (nonsmoker, light smoker (1–10 cigarettes/day), over 10 cigarettes/day including moderate (1/2-1 pack/d) and heavy (over 1 pack/d) smoker as well as ex-smoker are described in **Table 1.** Differences between groups were observed across variables. Light smokers were younger than nonsmokers and the group of moderate and heavy smokers (all p<0.001). Compared to light smokers and the group of moderate and heavy smokers, nonsmokers had lower BMI, body weight, waist circumference, systolic and diastolic blood pressure, triglyceride, uric acid (all p<0.001). In addition, ex-smokers were significantly older than current smokers (p<0.05). When compared with nonsmokers and current smokers including light, moderate and heavy smokers, higher fasting and postprandial blood glucose, hemoglobin A1c, sGOT, and the prevalence of diabetes were observed in ex-smokers (all p<0.001). The group of moderate and heavy smoker was older and had higher waist circumference, systolic blood pressure, fasting and postprandial blood glucose, hemoglobin A1c, triglyceride, sGPT, the prevalence of hyperlipidemia, diabetes than light smokers (all p<0.001). Finally, moderate and heavy smoker demonstrated higher CCS compared to non- and light smokers (p<0.001).

**Table 1:** Demographic and clinical characteristics according smoking habits.

		Current Smoker				
	Non-smoker	1~10 cigarettes/d	Over 10 cigarettes/d	Ex-smoker	p *	p ^$^ (or *X*^*2*^)
	2228 (71.7%)	449 (14.4%)	345 (11.1%)	87 (2.8%)		
***Baseline Demographics***						
Age (years)	49.85±9.82	47.5±9.38	50.67±9.32	51.66±8.83	<0.001*^$^	0.008^$^
Male gender, n (%)	1417 (63.5%)	421 (93.8%)	328 (95.0%)	86 (98.9%)	—	<0.001
BMI (kg/m^2^)	24.41±3.51	25.09±3.2	25.65±3.59	26.18±3.46	<0.001*^#^	<0.001*^#^
Height (cm)	164.33±13.05	169.74±6.61	169.15±6.57	168.01±5.85	<0.001*^#^	<0.001*^#^
Weight (kg)	66.45±12.84	72.5±11.44	73.56±12.08	74.14±12.11	<0.001*^#^	<0.001*^#^
Body fat (%)	23.48±10.59	23.66±7.77	24.19±8.57	23.29±10.01	.469	.594
Waist circumference (cm)	82.93±9.85	86.44±8.83	88.59±9.15	89.38±9.29	<0.001*^#^^$^	<0.001*^#^
Systolic blood pressure (mmHg)	122.94±17.24	120.33±15.61	125.55±16.22	126.84±16.54	<0.001*^#^^$^	0.087
Diastolic blood pressure (mmHg)	75.36±12.39	75.51±10.83	79.13±10.43	77.94±13.27	<0.001*^#^	0.001*
Pulse rate (bpm)	73.44±9.72	72.76±9.56	73.55±10.45	73.58±10.61	.369	.690
***Biochemical Data***						
Fasting glucose (mg/dL)	100.1±19.25	101.44±22.87	109.24±33.51	111.58±23.92	<0.001^#^^$^	<0.001*^#^^$^
Post-prandial glucose (mg/dL)	121.53±44.29	119.53±47.7	132.84±64.36	151.36±63.73	<0.001^#^^$^	<0.001^#^^$^
HbA1c (%)	5.8±0.78	5.84±0.93	6.15±1.08	6.22±1.05	<0.001^#^^$^	<0.001*^#^
Total cholesterol (mg/dL)	200.69±35.64	204.05±34.43	206.36±44.25	205.22±34.43	0.013^#^	0.013^#^
Triglyceride (mg/dL)	128.1±79.65	163.66±106.32	192.43±243.22	157.96±79.56	<0.001*^#^^$^	<0.001*
sGPT (U/dL)	28.99±21.63	33.71±30	37.69±39.23	40.08±40.39	<0.001^#^^$^	<0.001^$^
LDL (mg/dL)	129.41±32.37	133.94±31.12	133.58±33.03	136.28±30.4	0.008*	0.002*
HDL (mg/dL)	54.23±13.99	48.22±13.19	46.41±11.76	47.97±9.81	<0.001*^#^	<0.001*^#^
BUN (mg/dL)	12.16±3.9	12.46±3.43	12.67±3.67	12.63±3.36	.034	0.031*
Uric acid (mg/dL)	5.87±1.44	6.4±1.4	6.41±1.38	6.45±1.23	<0.001*^#^	<0.001*^#^
eGFR (ml/min/1.73m^2^)	82.82±15.45	82.21±13.68	81.53±14.82	78.89±13.53	.301	.033
***Medical History***						
Hyperlipidemia treatment, n (%)	146 (6.6%)	28 (6.2%)	35 (10.1%)	8 (9.2%)	—	0.072
History of hypertension, n (%)	347 (15.6%)	62 (13.8%)	87 (25.2%)	17 (19.5%)	—	<0.001
History of CVD, n (%)	85 (3.8%)	18 (4.0%)	25 (7.3%)	7 (8.1%)	—	0.009
History of diabetes, n (%)	102 (4.6%)	21 (4.7%)	37 (10.7%)	13 (14.9%)	—	<0.001
Regular exercise, n (%)	295 (13.2%)	66 (14.7%)	72 (20.9%)	8 (9.2%)	—	0.001
***Coronary Vascular Abnormality***						
CCS (n = 3084)	37.6±177.4	34.8±137.9	96.0±304.8	97.6±271.7	<0.001*	<0.001^#^^$^

p * ANOVA P value among groups (non-smoker, 1~10 cigarettes/day and over 10 cigarettes/day).

*:P<0.05 compared between group (Non-smoker) and group (1~10 cigarettes/day).

#:P<0.05 compared between group (Non-smoker) and group (over 10 cigarettes/day).

&:P<0.05 compared between group (1~10 cigarettes/day) and group (over 10 cigarettes/day).

p ^$^ ANOVA P value among groups (non-smoker, current smoker and quitted ex-smoker)

*:P<0.05 compared between group (Non-smoker) and group (current smoker).

#:P<0.05 compared between group (Non-smoker) and group (Ex-smoker).

&:P<0.05 compared between group (current smoker) and group (Ex-smoker).

Abbreviation: BMI, body mass index; sGPT serum glutamate pyruvate transaminase; eGFR, estimated glomerular filtration rate; HbA1C, glycosylated hemoglobin level; HDL, high-density lipoprotein; LDL, low-density lipoprotein.

### The association among smoking dose, duration and TAC

Among the smoking categories by smoking dose and duration, significant differences had been found in CCS and TAC related scores including TAC score, volume, density (p<0.001) and the presence of TAC (p<0.001). CCS and all TAC related scores were highest in the group of moderate and heavy smokers as well as the group of smoking duration of 3–5 years. Ex-smokers had higher CCS, TAC score, volume, density and prevalence of TAC than non- and light smokers but lower than the group of moderate and heavy smoker. (p<0.001). The groups of high-does and long-term smoking duration (>3 years) had higher prevalence of CCS and TAC than nonsmokers, low-does and short-term smokers. (p = 0.022 and p<0.001) (**Tables 2 and 3**).

**Table 2:** Distribution of TAC related score and prevalence of TAC by smoking dose.

		Current smoker			
Smoking Dose	Non-smoker	1~10 cigarettes/day	Over 10 cigarettes/day	Ex-smoker	p (ANOVA or *X*^*2*^)
	2228 (71.7%)	449 (14.4%)	345 (11.1%)	87 (2.8%)	
**TAC score**	38.6±158.8	50.8±266.8	397.3±1987.2*	133.1±575.5*	<0.001
**TAC volume (mm**^**3**^**)**	32.9±132.0	42.3±218.0	274.9±1148.6*	112.0±466.2*	<0.001
**TAC density (HU)**	41.9±97.9	36.8±91.2	67.1±128.7*	61.7±110.6*	<0.001
**CCS**	35.9±158.4	34.8±137.9	96.0±304.8*†	97.6±271.7*†	<0.001
**Presence of thoracic Calcification (%)**					
** No**	2006 (92.9%)	319 (91.9%)	196 (87.1%)	29 (90.6%)	0.022
** Yes**	154 (7.1%)	28 (8.1%)	29 (12.9%)	3 (9.4%)	

*P<0.05 compared between non-smoker group

†P<0.05 compared to 1~10 cigarettes/day group.

**Table 3:** Distribution of TAC related score and prevalence of TAC by smoking duration.

		Current smoker					
Smoking duration	Non-smoker	<1 year	1–3 years	3–5 years	5–10 years	Over 10 years	p (pearson or *X*^*2*^)
**TAC score**	35.07±146.39	4.34±13.66	18.94±63.64	392.95±789.70	253.31±826.09	154.89±721.56	<0.001*
**TAC volume (mm**^**3**^**)**	30.33±123.66	4.39±13.68	18.25±60.26	328.75±655.40	160.88±538.09	129.9±599.86	<0.001*
**TAC density (HU)**	40.79±96.61	32.46±85.92	26.49±73.77	92.57±142.15	48.06±114.36	52.64±113.54	0.013*
**CCS**	31.84±138.80	32.36±72.36	129.20±364.02	246.88±535.71	233.55±781.92	94.85±325.07	<0.001
**Presence of thoracic Calcification (%)**							
** No**	1948 (89.80%)	4 (100%)	6 (85.7%)	8 (72.7%)	18 (72%)	227 (73.5%)	<0.001
** Yes**	222 (10.20%)	0 (0%)	1 (14.3%)	3 (27.3%)	7 (28%)	82 (26.5%)	

*:P<0.05 compared between group (Non-smoker) and group (current smoker).

In multivariate regression analyses, moderate (1/2-1 pack /cigarettes/d) and heavy (over 1 pack/d) smokers were associated with higher TAC score, TAC volume, TAC density, and the presence of calcification independent of age, gender. When BMI and clinical cardiovascular risk factors were further added in these models, all TAC-related scores remained associated with moderate and heavy smokers except the association of moderate smoker and TAC density. Meanwhile, light smokers (1-10cigerettes/d) were not associated with TAC score, TAC volume and TAC density but remained association with presence of calcification in multi-variate regression analyses. (**Table 4**). Total pack-years of current smoker are strongly associated with all TAC-related scores and presence of TAC after adjustment for age, gender and cardiovascular risk factors (**Table 5**). In multivariate analysis of smoking duration, TAC related scores were positively associated with all groups of smoking duration > 3 years in all models (p<0.001). No association was observed in the groups who have been smoking < 3 years. In addition, the group who have been smoking over 10 years tend to calcification of thoracic aorta after adjustment for age, gender and cardiovascular risk factors. (p<0.001; **S1 Table**). By mutually further adjusting for TAC density (HU) (in TAC volume model) and TAC volume (mm^3^) (in TAC density model) in multivariate analysis, moderate (1/2-1 pack /cigarettes/d) and heavy (over 1 pack/d) smokers, along with larger pack-years of smoking remained independently associated with high TAC volume, rather than TAC density **(Tables 4 and 5, respectively).**

**Table 4:** The Association of smoking doses (cigarettes/day) with TAC related score after adjustment for age, gender, and cardiovascular risk factors.

	Smoking dose	Uni-variate model		Multi-variate Models					
				Model 1		Model 2		Model 3	
		**Coef [95%CI]**	**p**	**Coef [95%CI]**	**p**	**Coef [95%CI]**	**p**	**Coef [95%CI]**	**p**
**TAC score**	**1~10 cigarettes/d**	3.78[-47.23, 54.78]	0.885	46.33[-3.70, 96.36]	0.07	27.51[-23.70, 78.72]	0.292	—	—
	**1/2~1 pack cigarettes/d**	247.93[158.30, 337.56]	<0.001	254.09[167.59, 340.59]	<0.001	185.85[96.18, 275.53]	<0.001	—	—
	**Over 1 pack cigarettes/d**	511.14[448.56, 573.73]	<0.001	484.88[423.89, 545.86]	<0.001	475.34[412.68, 537.99]	<0.001	—	—
**TAC volume**	**1~10 cigarettes/d**	3.80[-37.56, 45.16]	0.857	39.64[-0.80, 80.08]	0.055	22.92[-18.15, 64.00]	0.274	34.23[-16.6, 85.06]	0.19
	**1/2~1 pack cigarettes/d**	197.81[125.13, 270.48]	<0.001	203.06[133.13, 272.98]	<0.001	144.62[72.70, 216.54]	<0.001	137.45[67.01, 207.9]	<0.001
	**Over 1 pack cigarettes/d**	399.17[348.42, 449.92]	<0.001	377.18[327.89, 426.48]	<0.001	362.92[312.66, 413.17]	<0.001	389.5[303.7, 475.3]	<0.001
		—	—						
**TAC density**	**1~10 cigarettes/d**	-7.66[-21.48, 6.16]	0.277	9.67[-2.73, 22.08]	0.126	10.67[-2.11, 23.45]	0.102	8.06[-1.00, 17.13]	0.081
	**1/2~1 pack cigarettes/d**	45.22[20.94, 69.50]	<0.001	45.78[24.34, 67.23]	<0.001	35.47[13.10, 57.85]	0.002	9.43[-3.17, 22.03]	0.14
	**Over 1 pack cigarettes/d**	63.07[46.11, 80.02]	<0.001	48.22[33.10, 63.33]	<0.001	48.96[33.33, 64.60]	<0.001	-0.87[-16.4, 14.67]	0.91
		—	—						
		**Odds ratio [95%CI]**	**p**	**Odds ratio[95%CI]**	**p**	**Odds ratio[95%CI]**	**p**	**Odds ratio[95%CI]**	**p**
**Presence of Calcification**	**1~10 cigarettes/d**	1.77[1.22, 2.56]	0.003	2.27[1.51, 3.40]	<0.001	2.17[1.40, 3.37]	0.001	—	—
	**1/2~1 pack cigarettes/d**	3.94[2.36, 6.57]	<0.001	4.00[2.25, 7.10]	<0.001	3.00[1.60, 5.63]	0.001	—	—
	**Over 1 pack cigarettes/d**	3.71[2.61, 5.27]	<0.001	2.79[1.90, 4.08]	<0.001	2.54[1.67, 3.87]	<0.001	—	—

Reference group was non-smoker. CI: confidence interval. Abbreviations as Table 1.

Model 1: smoking dose, age, sex; Model 2: smoking dose, age, sex, BMI, SBP, pulse rate, fasting glucose, total cholesterol, HDL, eGFR, HTN, CVD, and Diabetes history; Model 3: model 2 plus TAC density in TAC volume model, model 2 plus TAC volume in TAC density model.

**Table 5:** The Association of total pack-year of current smoker with TAC related score after adjustment for age, gender, and cardiovascular risk factors.

Outcome	Uni-variate model		Multi-variate Models					
	(Per 1 pack-year +)		(Per 1 pack-year +)					
	crude model		Model 1		Model 2		Model 3	
	**Coef [95%CI]**	**p**	**Coef [95%CI]**	**p**	**Coef [95%CI]**	**p**	**Coef [95%CI]**	**p**
**TAC score**	25.16[21.95, 28.38]	<0.001	25.44[22.29, 28.58]	<0.001	23.71[20.42, 27.01]	<0.001	—	—
**TAC volume**	19.87[17.26, 22.48]	<0.001	20.09[17.54, 22.63]	<0.001	18.41[15.78, 21.05]	<0.001	14.37[2.76, 25.97]	0.015
**TAC density**	2.45[1.59, 3.32]	<0.001	2.41[1.64, 3.19]	<0.001	2.32[1.51, 3.13]	<0.001	0.81[-1.00, 2.63]	0.38
	**Odds ratio [95%CI]**	**p**	**Odds ratio [95%CI]**	**p**	**Odds ratio [95%CI]**	**p**	**Odds ratio [95%CI]**	**p**
**Presence of Calcification**	0.014[0.011, 0.017]	<0.001	0.013[0.010, 0.016]	<0.001	0.012[0.009, 0.015]	<0.001	—	—

CI: confidence interval. Abbreviations as Table 1.

Model 1: smoking dose, age, sex; Model 2: smoking dose, age, sex, BMI, SBP, pulse rate, fasting glucose, total cholesterol, HDL, eGFR, HTN, CVD, and Diabetes history; Model 3: model 2 plus TAC density in TAC volume model, model 2 plus TAC volume in TAC density model.

### The association among smoking exposure history/patterns and TAC

**Fig 2:**
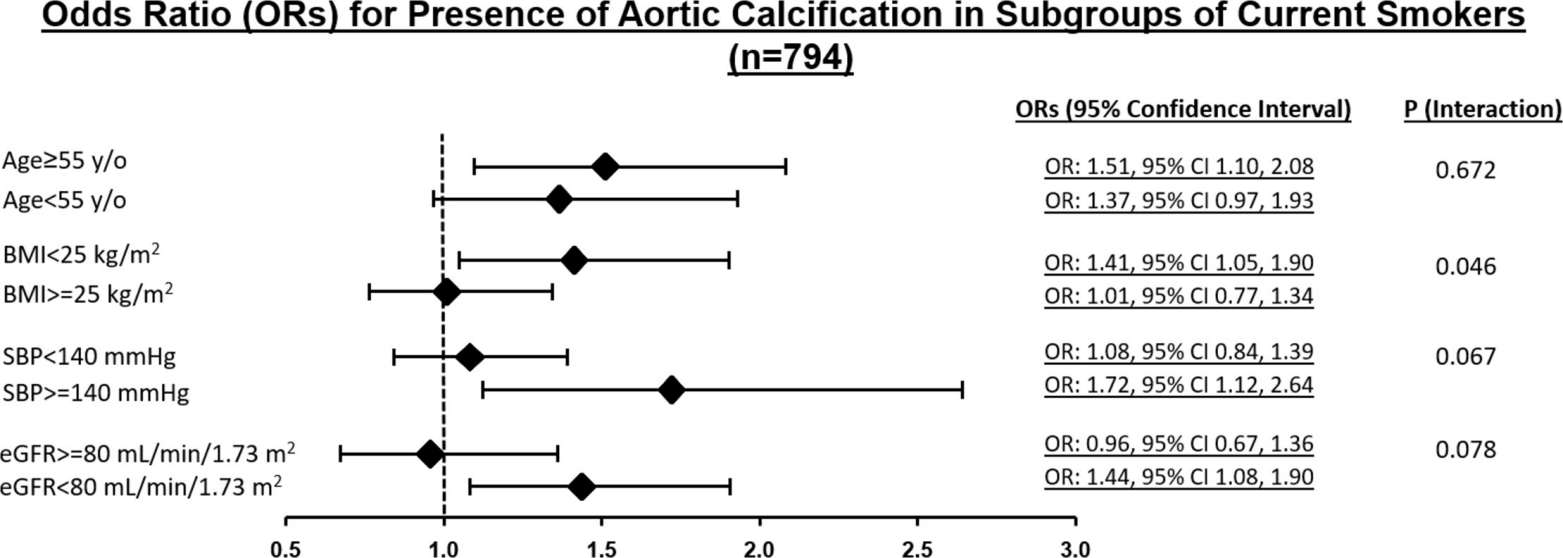
Factors mediating modifying effects of aortic calcification and active smoking. Presence of thoracic aortic calcification with active smoking showed no effect modifications by age (<55, ≥55 years [median: 49 years in current study]), though was modified by BMI (<25, ≥25 kg/m^2^, p _interaction_: 0.046), and showed marginal differential associations with SBP (<140, ≥140 mmHg, p _interaction_: 0.067) and renal function in terms of eGFR (<80, ≥80 mL/min/1.73 m^2^, p _interaction_: 0.078).

**Fig 3:**
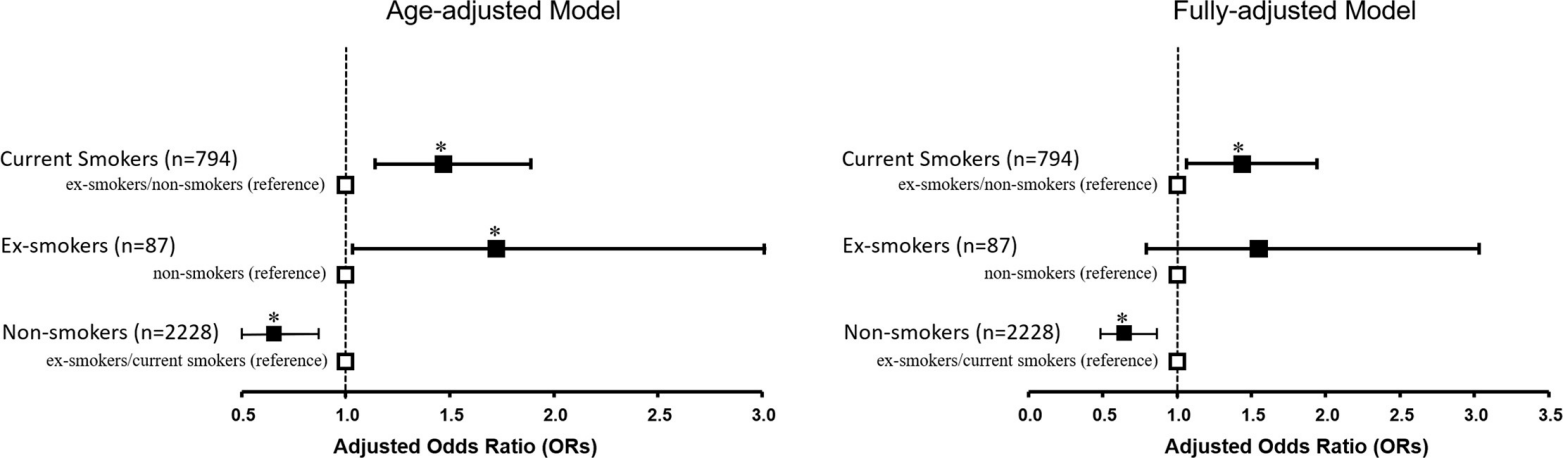
Risk of aortic calcification among subjects with differential smoking exposure patterns in present work. Markedly increased risk of aortic calcification in current smokers in contrary to those who never or quitted from smoking (ex-smokers) after adjusting for age and relevant clinical cardiovascular risks (ex-smokers and non-smokers as reference group). Subjects who quit smoking (ex-smokers) showed significantly attenuated aortic calcification in age-adjusted and fully adjusted models (non-smokers as reference). Instead, subjects who never smoke showed consistently reduced risk of aortic calcification, even after fully adjustment (Current and ex-smokers as reference group). *denotes p<0.05.

We further examined whether there existed differential associations (modifying effects) between presence of aortic calcification and active smoking by various key clinical demographics information including age (≥55, <55), BMI (≥25, <25), systolic blood pressure (≥130, <130mmHg), and eGFR (set at 80 mL/min/1.73 m^2^) (**Fig 2**). Lean body size showed significantly more pronounced effects of aortic calcification in active smokers (p _interaction_: 0.046) with a borderline trend that higher blood pressure (≥140mmHg) and worse renal function (<80 mL/min/1.73 m^2^) also modified associations between active smoking and aortic calcification (p _interaction_: 0.067 and 0.078, respectively). These modifying effects became attenuated after further multi-variate adjustment. As age alone remained highly associated with the presence of aortic calcification in current study (OR: 11.8, [95% CI: 11.6–11.9 per decade increase], p<0.001), we further examined the associations of different smoking exposure patterns/history (such as current smokers, Ex-, and Non-smokers) and TAC in current study. Subjects as categorized as current smokers showed consistently higher risk for TAC existence when compared to Non- and Ex-smokers after accounting for age and all clinical co-variates (adjusted OR: 1.48 [95% CI: 1.15–1.90], P = 0.002; adjusted OR: 1.47 [95% CI: 1.10–1.89], P = 0.009 for age and fully-adjusted models, respectively) **(Fig 3)**. As ex-smokers showed independent association with TAC presence after age adjustment (adjusted OR: 1.83 [95% CI: 1.02–3.30], P = 0.044), such association was attenuated in fully adjusted models (P = 0.20). Instead, there was consistently protective effect in non-smokers, with age and relevant clinical co-variates adjusted (adjusted OR: 0.64 [95% CI: 0,50–0.,82], P<0.001; adjusted OR: 0.65 [95% CI: 0.48–0.86], P = 0.003, respectively).

## Discussion

In this retrospective cross-sectional study, we investigated the relationship between smoking habits and calcified plaque burden in the thoracic aorta in over 3,000 patients enrolled at a single center. The main findings of this study are summarized as follow: First, moderate and heavy current smokers had significantly higher TAC score, TAC volume and TAC prevalence than light current smokers, non-smokers, and ex-smokers; Second, all smokers with a longer smoking duration (≥ 3 years) had significantly higher TAC score, TAC volume, TAC density and TAC prevalence than all smokers with a shorter smoking duration (< 3 years); Third, all the TAC related scores tended to increase proportionally with smoking dose, pack-year and smoking duration in the current smokers after adjusting for the baseline clinical risk factors.

In our study, multi-detector computed tomography (MDCT) was used to measure the thoracic aortic calcium burden in terms of the TAC score, plaque volume and plaque density (in continuous HU value). In the past, researchers mainly focused on the relationship of Agatston score and cardiovascular diseases.[17,18] The Agatston method calculates the total calcium score based on the number, area, and category of the range of CT number (in Hounsfield Unit) of the calcified lesions in the coronary arteries.[18] Despite its wide adoption in assessing the risk of future cardiovascular events, the Agatston score cannot represent all aspects of calcified plaque burden. The prognostic values of the calcified plaque volume and density in the coronary arteries and aorta have been recently investigated. Forbang et al reported in the multi-ethnic study of atherosclerosis (MESA) that a number of CVD risk factors were associated with higher volume but lower density of abdominal aortic calcified plaques.[13] Furthermore, Bellasi et al showed that in hemodialysis patients, increased density of coronary calcified plaques is an independent predictor of all-cause of mortality in hemodialysis patients.[19] Such inverse correlation could be related to the fact that plaque with thicker membrane cap and higher calcium content may be less prone to rupture [20] The vulnerable plaque was characterized as thin-cap fibroatheroma with thin membrane cap, large necrotic or lipid core and may lead to plaque rupture and subsequent thrombosis.[21] Previous coronary CTA studies have demonstrated lower rates of adverse CVD in patients with primary calcified plaques, compared to those with noncalcified and mixed (calcified and noncalcified) plaques.[22,23] In line with prior reports, we also demonstrated overall greater coronary calcification burden in more heavy smokers in our current work. On the other hand, in dialysis patients with advanced chronic kidney disease, calcification in the media is more frequently compared to calcification in the intima associated with atherosclerotic plaques in general populations[16]. However, those studies have the limitation by using a 4-point scale rather than a continuous HU scale to assess plaque density which could limit power and may not detect significant correlations. In addition, TAC related scores may play different roles in different races. As shown in the MESA study, Hispanic Americans, African Americans and Chinese Americans had lower abdominal aortic calcified plaque volume, but higher plaque density and slower rate of TAC progression compared to European Americans.[13] Therefore, compared to the concrete scientific evidences of Agatston score, the association between TAC related scores and CV risk in a large Chinese population remains to be demonstrated and addressed in the future studies.

Tobacco smoking is one of the major preventable causes of stroke, CVD, and ischemic heart disease [24], and approximately 35–40% of CVD-related deaths are attributed to the effects of smoking.[25] It is particularly important to conduct in-depth analysis of the impact of smoking on TAC in Chinese smokers. Over 50% of the smokers in the world resides in Asian countries with the smoker in those countries continues to as opposed to developed countries.[26] Although cigarette smoke comprises a number of cardiotoxins [27], the underlying mechanism between smoking and cardiovascular diseases remains loosely understood. Among these toxic chemicals, nicotine, carbon monoxide and oxidizing agents are most related to the pathogenesis of cardiovascular diseases [28]. Smoking is also associated with dose-related impairment of endothelium-dependent arterial dilation and increased intima-media thickness of the carotid arteries [29,30]. Additionally, the endothelial damage and inflammation caused by free radicals and nicotine carried by cigarette smoke into the blood stream suggested that plaque formation and calcification in the walls of small and large vessels could be associated with the number of cigarettes smoked. Our findings are consistent with those of previous studies in the strong association of smoking and atherosclerosis [31–33]. We demonstrated that light smokers exhibited significantly lower TAC score, density and volume compared to non-smokers as well as a longer smoking duration (≥ 3 years) had significantly higher TAC score, density and volume and TAC prevalence. Meanwhile, moderate and heavy smokers had significantly higher plaque density compared to light smokers which indicates the progression of arterial calcification. It suggested that smoking dose and duration have continuing effects rather than simply a triggering effect which may be subsided after applying appropriate control measures [34]. Further, we observed stronger associations between moderate to heavy smokers in terms of higher number of daily smoking behavior or longer use with TAC volume even after adjusting for lesion density, indicating that increased vascular volume per se may likely play as central key roles in this pathological process. While smoking cessation is associated with a lower risk of CVD, it has been reported that smoking cessation reduced the risk of aortic arch calcification only for males ex-smokers who had light smoking habits [7]. This finding is consistent with our findings of the lower TAC related scores and prevalence of aortic calcification of ex-smoker than the group of moderate and heavy smoker.

It is noteworthy to mention the several limitations associated to this study. Firstly, only non-contrast enhanced CT images were available for our retrospective analysis, therefore non-calcified atherosclerotic plaques in the aorta could not be assessed; Secondly, ECG-gated heart CT study does not cover the level of aortic arch which is the typical region with atherosclerotic change. Thirdly, smoking behavior was largely confined to male gender in our current study cohort which may limited a proper sex-based analysis, but this situation could be applicable to population-based situations and therefore our results could still inform a future study on sex differences; Forth, our study design is a retrospective cross-sectional study, which is useful to determine the prevalence of disease but not the prognostic value of each smoking habit (such as the number of packs per day).

In conclusion, the findings of our retrospective analysis on 3109 patients suggest a strong relationship between the cigarette dose and the TAC burden in both current and ex-smokers. The underlying pathophysiological mechanisms as well as the prognostic values of TAC related scores should be further explored.

## Supporting information

S1 Table The Association of smoking duration with TAC related score after adjustment for age, gender, and cardiovascular risk factors. (DOCX)
