# Supplementary material for: Associations of cigarette smoking and burden of thoracic aortic calcification in asymptomatic individuals: A dose-response relationship
Source: PLoS One. 2020 Jan 9;15(1):e0227680. doi: 10.1371/journal.pone.0227680 (PMC6952096; doi:10.1371/journal.pone.0227680)
Supplement: S1 Table — (DOCX) [file pone.0227680.s001.docx]

**S1 Table. The Association of smoking duration with TAC related score after adjustment for age, gender, and cardiovascular risk factors**

|  | **Smoking year** | **Uni-variate Model** | | **Multi-variate Models** | | | | | |
| --- | --- | --- | --- | --- | --- | --- | --- | --- | --- |
|  |  |  | | **Model 1** | **Model 2** | | **Model 3** | | |
|  |  | **Coef [95%CI]** | **p** | **Coef [95%CI]** | **p** | **Coef [95%CI]** | **p** | **Coef [95%CI]** | **p** |
| **TAC score** | **< 1 year** | -35.07 [-404.53, 334.39] | 0.852 | 26.71 [-326.56, 379.98] | 0.882 | 34.91 [-318.44, 388.25] | 0.846 | 65.44 [-277.43, 408.31] | 0.708 |
|  | **1- 3 years** | -32.98 [-312.46, 246.50] | 0.817 | -3.96 [-271.15, 263.23] | 0.977 | -2.29 [-269.42, 264.84] | 0.987 | -33.03 [-292.12, 226.07] | 0.803 |
|  | **3-5 years** | 833.40 [610.25, 1056.55] | <0.001 | 801.22 [587.86, 1014.58] | <0.001 | 805.93 [592.54, 1019.33] | <0.001 | 828.06 [598.69, 1057.44] | <0.001 |
|  | **5-10 years** | 679.33 [527.80, 830.85] | <0.001 | 698.63 [553.76, 843.50] | <0.001 | 705.23 [560.14, 850.31] | <0.001 | 689.70 [522.66, 856.74] | <0.001 |
|  | **Over 10 years** | 196.79 [151.38, 242.20] | <0.001 | 205.49 [162.07, 248.91] | <0.001 | 212.98 [168.49, 257.46] | <0.001 | 190.36 [144.29, 236.42] | <0.001 |
| **TAC volume** | **< 1 year** | -30.33 [-331.30, 270.65] | 0.843 | 21.73 [-265.10, 308.56] | 0.882 | 28.63 [-258.25, 315.51] | 0.845 | 51.55 [-224.25, 327.35] | 0.714 |
|  | **1- 3 years** | -27.35 [-255.02, 200.32] | 0.814 | -2.90 [-219.85, 214.04] | 0.979 | -1.50 [-218.38, 215.39] | 0.989 | -25.80 [-234.22, 182.61] | 0.808 |
|  | **3-5 years** | 695.16 [513.38, 876.95] | <0.001 | 668.05 [494.82, 841.28] | <0.001 | 672.02 [498.76, 845.27] | <0.001 | 682.94 [498.44, 867.45] | <0.001 |
|  | **5-10 years** | 408.83 [285.39, 532.26] | <0.001 | 425.10 [307.47, 542.72] | <0.001 | 430.65 [312.85, 548.44] | <0.001 | 345.99 [211.63, 480.36] | <0.001 |
|  | **Over 10 years** | 163.21 [126.22, 200.20] | <0.001 | 170.53 [135.28, 205.79] | <0.001 | 176.84 [140.72, 212.95] | <0.001 | 157.15 [120.10, 194.21] | <0.001 |
| **TAC density** | **< 1 year** | -40.79 [-139.56, 57.97] | 0.418 | -13.76 [-100.35, 72.84] | 0.755 | -11.49 [-98.10, 75.11] | 0.795 | -8.74 [-92.30, 74.81] | 0.837 |
|  | **1- 3 years** | -13.61 [-88.32, 61.10] | 0.721 | -0.91 [-66.41, 64.58] | 0.978 | -0.45 [-65.92, 65.02] | 0.989 | -5.11 [-68.25, 58.03] | 0.874 |
|  | **3-5 years** | 145.46 [85.81, 205.11] | <0.001 | 131.38 [79.08, 183.68] | <0.001 | 132.68 [80.38, 184.98] | <0.001 | 144.82 [88.92, 200.71] | <0.001 |
|  | **5-10 years** | 56.96 [16.46, 97.47] | 0.006 | 65.41 [29.90, 100.92] | <0.001 | 67.24 [31.68, 102.79] | <0.001 | 52.72 [12.02, 93.43] | 0.011 |
|  | **Over 10 years** | 13.17 [1.03, 25.31] | 0.033 | 16.98 [6.33, 27.62] | 0.002 | 19.05 [8.14, 29.95] | 0.001 | 18.46 [7.24, 29.69] | 0.001 |
|  |  | **Odds ratio [95%CI]** | **p** | **Odds ratio [95%CI]** | **p** | **Odds ratio [95%CI]** | **p** | **Odds ratio [95%CI]** | **p** |
| **Presence of Calcification** | **< 1year** | 1 (reference) |  | 1 (reference) |  | 1 (reference) |  | 1 (reference) |  |
|  | **1- 3years** | 1.46 [0.18, 12.20] | 0.725 | 1.83 [0.19, 17.39] | 0.600 | 1.76 [0.18, 17.51] | 0.630 | 0.97 [0.08, 11.68] | 0.984 |
|  | **3-5years** | 3.29 [0.87, 12.49] | 0.080 | 2.58 [0.56, 11.95] | 0.224 | 2.38 [0.52, 11.00] | 0.266 | 2.59 [0.50, 13.43] | 0.258 |
|  | **5-10 years** | 3.41 [1.41, 8.26] | 0.007 | 4.56 [1.61, 12.89] | 0.004 | 4.08 [1.46, 11.41] | 0.007 | 3.21 [0.90, 11.47] | 0.072 |
|  | **Over 10 years** | 3.17 [2.37, 4.23] | <0.001 | 4.29 [3.11, 5.90] | <0.001 | 3.73 [2.69, 5.17] | <0.001 | 3.52 [2.46, 5.05] | <0.001 |

Reference group was non-smoker. CI: confidence interval. Abbreviations as Table 1.

Model 1: smoking years, age

Model 2: smoking years, age, sex

Model 3: smoking years, age, sex, BMI, SBP, pulse rate, fasting glucose, total cholesterol, HDL, eGFR, HTN, CVD, and Diabetes history.
